# From pills to pounds: prospective insights into the weight effects of antidepressant use

**DOI:** 10.1192/j.eurpsy.2026.10330

**Published:** 2026-07-31

**Authors:** R. Musil

**Affiliations:** 1 Psychosomatic, Oberberg Fachklinik Bad Tölz, Bad Tölz; 2 Psychiatry and Psychotherapy, LMU Munich, Munich, Germany

## Abstract

**Background:**

Antidepressants are essential for treating major depressive disorder, yet weight gain remains a significant side effect. It contributes to metabolic morbidity, reduced treatment adherence, and long-term cardiovascular risks.

**Objectives:**

This review provides an overview of the prevalence, pharmacological mechanisms, and clinical implications of antidepressant-associated weight gain, alongside prevention and management strategies.

**Methods:**

A selective literature review was conducted, analyzing randomized controlled trials, meta-analyses, and large cohort studies focusing on compound-specific weight changes.

**Results:**

Weight gain varies significantly by substance. It is most pronounced with tricyclic antidepressants, mirtazapine, and maprotiline. Among SSRIs, paroxetine is the most weight-promoting, while fluoxetine is the most favorable. Conversely, bupropion and agomelatine are associated with weight neutrality or modest loss.

While short-term treatment may cause initial weight loss, long-term use often results in gradual gain. Mechanisms include H1, M1, and 5-HT2C receptor antagonism and modulation of appetite-regulating neuropeptides (leptin, ghrelin). Notably, a 5% weight increase within the first month correlates with a 5.5-fold increased risk of metabolic syndrome (MetS) by the sixth month. Newer agents like vortioxetine and vilazodone show promise in minimizing these metabolic burdens.

**Conclusions:**

Weight gain is a critical adverse effect that requires proactive management. Treatment should integrate careful drug selection, early monitoring, and emerging therapies such as GLP-1 receptor agonists. Balancing psychiatric efficacy with metabolic safety is essential to improve adherence and restore the “whole person.”

**Disclosure of Interest:**

None Declared

